# The Book World of Medicine and Science

**Published:** 1900-09-01

**Authors:** 


### The Book World of Medicine and Science.

Appendicitis. By A. H. Tubby, M.S., F.R.C.S., Surgeon to Westminster Hospital, and to the National Orthopædic Hospital. Senior Surgeon to the Evelina Hospital for Children. Crown 8vo., 100 pp. (London: Ballière, Tindall, and Cox. Price 2s. 6d. net.)

This volume is the third of the "Medical Monographs" which are being issued so as to present a concise survey of subjects of everyday interest to students and practitioners, and it deals with the lesion known as appendicitis. It is clearly and well written, and contains a good *resumé* of the present opinions concerning the disease. It is written by a surgeon, but by one who knows well how to weigh evidence, and therefore no extreme statements are to be found in its pages. After describing the normal anatomy of the vermiform appendix and its annexa, the author discusses the morbid anatomy of inflammation of the organ. While agreeing that there is a possibility of true typhlitis, he considers it a very rare condition, and he is also sceptical as to the existence of "appendicular colic." That inflammation of the appendix is generally associated with superjacent peritonitis is a recognised fact at the present day, and this without any actual perforation of the tube. In reviewing the signs and symptoms of the affection, the writer lays stress upon pain, the temperature, and the pulse and respiration rate, together with rigidity, tenderness, and the presence of a tumour. There is an excellent account of the examination of a patient for the detection of the appendix in a case of relapsing appendicitis, and of the errors into which the surgeon may fall in diagnosing other conditions present as indicative of an enlarged appendix. These are the existence of a psoas parvus, local contraction of muscular tissue in the abdominal wall, or indurated omentum. The complications and sequelæ are well discussed, as also is diagnosis. Prognosis is rightly accorded a chapter to itself, and is reviewed in a thorough manner. Some disappointment, however, occurs when the last chapter, that on treatment, is reached. It only occupies barely ten pages, and deserves fuller handling. The non-operative treatment is discussed clearly, particularly the question as to use or non-use of opium and purgatives. But it would have been more satisfactory could the author have given greater detail as to the actual operative procedure in instances of localised abscess, which has in many cases to be evacuated with but little assistance. The book can be highly recommended to every practitioner as one likely to give considerable help in dealing with the all too frequent cases of appendicitis.

Practical Text Book of Midwifery for Nurses and Students. By Robert Jardine, M.D.Edin., M.R.C.S. Eng., F.F.P. and S.Glas. (Edinburgh: William F. Clay. 1899. With 36 illustrations. Price 6s.)

That this book is written for the use of nurses or midwives rather than of medical students is made manifest by the introductory chapter, which is devoted to one of those elementary descriptions of the general anatomy and physiology of the body with which we have now become so familiar in every manual for nurses; and not unnaturally the first question which will arise in the minds of any medical man who takes it up will be whether it is wise to carry the subject of midwifery so far as is done in its pages before an audience which is presumed to be absolutely ignorant of the very principles of anatomy and physiology, and is probably therefore incapable of understanding the bearing of one-tenth part of what the book contains. If, however, we take it, as is probably the case, that this first chapter was merely put in for the sake of completeness to round off the volume, and if we decline to regard it as indicating the mental and educational standard of those for whom the book is intended, we may say at once that the work is admirably designed and carried out as a teaching handbook for the midwife. Just as a nurse, who of course never has herself to treat a case of pneumonia, still wants to know, as she would say, "all about it," so a midwife, notwithstanding that her duty absolutely forbids her undertaking the management of a transverse presentation, still hankers after knowledge on the subject, and is probably none the worse for having it. If a midwife's knowledge were to be limited to the practical duties of her daily life, no doubt a little book of precepts how to conduct a normal labour and when to send for help would be all that would be wanted. But the human mind is not made that way, it "wants to know, you know"; and if we are to tempt the women with the better minds to take up the work of midwifery, we must encourage them to know far more than they ever need practice. And in teaching this wider knowledge, while at the same time telling where the limit in practice must be drawn, Dr. Jardine's book will be found an admirable guide and a reliable mentor. It is readable, well printed, sufficiently well illustrated, and the advice it gives may be followed with confidence.

Instructions for the Prevention of Malarial Fever for the Use of Residents in Malarial Places. (Second edition. Liverpool: The University Press. 1900.)

In this pamphlet, issued by the Liverpool School of Tropical Diseases, the mosquito theory is explained in popular language, and rules are laid down in accordance with its teaching for preventing the disease. The facts are well stated, and the teaching is fully in accordance with them. The rub comes when the teaching has to be put into practice. As it stands on paper it is simplicity itself. "We must (1) avoid being bitten by mosquitos as much as possible, (2) kill mosquitos or their larvæ, especially anopheles, or prevent them breeding round our houses."

### NOTES ON BOOKS.

The holiday season has led to the multiplication of guide books and travellers' manuals, some of which, and some be it said of the most useful, are merely advertisements of places and of ways of getting there. Such are the little pamphlets on Aldeburgh and on Hunstanton issued by the Great Eastern Railway. They are both cram full of information and are nicely illustrated, and as they both make us wish we were there, we suppose they do just what they were wanted to do. Rather on a different plane must be mentioned the sixpenny "Tourist Guide to the Continent," edited by Percy Lindley, and issued by the same company. It is a well illustrated and really a very interesting guide book to a long string of places chiefly in Holland and Belgium, Germany and Switzerland, which may be reached viâ the Hook of Holland. We have many a time travelled by that route, and can fully endorse what is said about its comfort.

